# Data on assessment of safety and tear proteome change in response to orthokeratology lens – Insight from integrating clinical data and next generation proteomics

**DOI:** 10.1016/j.dib.2020.105186

**Published:** 2020-01-28

**Authors:** Jimmy Sung-Hei Tse, Thomas Chuen Lam, Jimmy Kai-Wai Cheung, Ying-Hon Sze, Tsz-King Wong, Henry Ho-Lung Chan

**Affiliations:** Laboratory of Experimental Optometry, Centre for Myopia Research, School of Optometry, The Hong Kong Polytechnic University, Hong Kong

**Keywords:** Tears, Orthokeratology, SWATH, Myopia

## Abstract

Breath-O™ Correct Ortho-K lenses are newly designed ortho-K lenses which are made from a silicon and fluoride containing methacrylate compound. This compound is said to be more flexible, durable and less likely to break compared to traditional Ortho-K lenses. The special design of this Ortho-K lens can reshape the corneal profile to induce temporary myopic reduction while producing beneficial peripheral hyperopic defocus for myopia control. To evaluate the safety and ocular surface responses of overnight Ortho-K wear over 1 and 3 months using this new type of material, we evaluated the clinical parameters (corneal integrity, corneal biomechanics, corneal endothelial health, non-invasive keratographical break-up time) and profiled the change of global tear proteome on healthy young subjects using next generation proteomics (SWATH-MS). The acquired mass spectrometric data were processed and analyzed using a cloud based Oneomics™ bioinformatic platform. All raw data generated from Information-dependent acquisition (IDA) and SWATH acquisitions were accepted and published in the Peptide Atlas public repository for general release (http://www.peptideatlas.org/PASS/PASS01367).

**Table:** Specifications Table

Subject	Biology
Specific subject area	The effect on tear proteome with orthokeratology lens made of a new rigid lens material (after 1 month and 3 months wear)
Type of data	Table, Figure
How data were acquired	Quadrupole Time-of-Flight TripleTOF® 6600 mass spectrometer (SCIEX); SWATH Mass Spectrometry, Searched against UniProt database (Homo Sapiens, organism ID: 9606)
Data format	Raw and Analyzed
Parameters for data collection	Orthokeratology lens wear for 1 and 3 months (compared to its age matched controls)
Description of data collection	Tears proteins were collected on 1 and 3 months after Ortho-K lens wearing (n = 12 at each time point) and age matched control subjects without lens wear (n = 12). Tears proteome libraries were generated, and differential proteins were identified and quantified using SWATH mass spectrometry (SWATH-MS).
Data source location	Centre for Myopia Research, School of Optometry, The Hong Kong Polytechnic University, Kowloon, Hong Kong
Data accessibility	Repository name: Peptide Atlas public repository Data identification number: PASS01367 Direct URL to data: http://www.peptideatlas.org/PASS/PASS01367
Related research article

**Table:** 

**Value of the Data** • This data included the tear database consisting of tears proteins during Ortho-K lenses wearing for a short (1 month) and longer period (3 months) of time which will be useful for clinical tears study involving the effect orthokeratology lenses. To the best of our knowledge, there is no similar report on this topic. • The effects of overnight Breath-O™ Correct Ortho-K lenses wearing on tear proteome, which may be used as an objective molecular evaluation of the ocular conditions in response to the lens wear. • The identified and quantified differential expressed tears proteins from SWATH-MS after 1 and 3 months of newly designed Ortho-K lenses wearing might reveal underlying changes on how orthokeratology lens affects the eye to provide insights on suitable materials choices for the development of Ortho-K lenses.

## 1 Data description

**Fig. 1:**
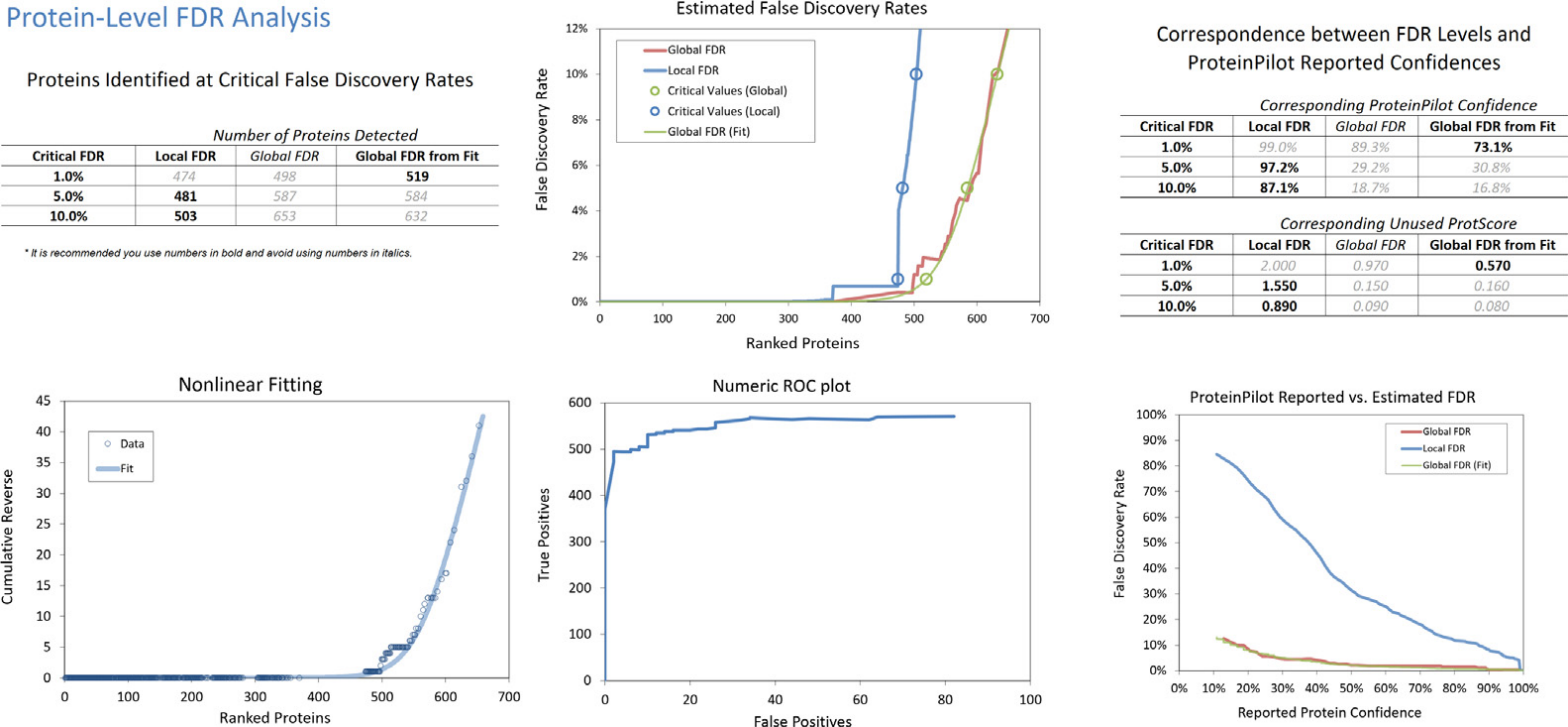
FDR analysis of the combined IDA ion library of tears at protein level from ProteinPilot software.

**Fig. 2:**
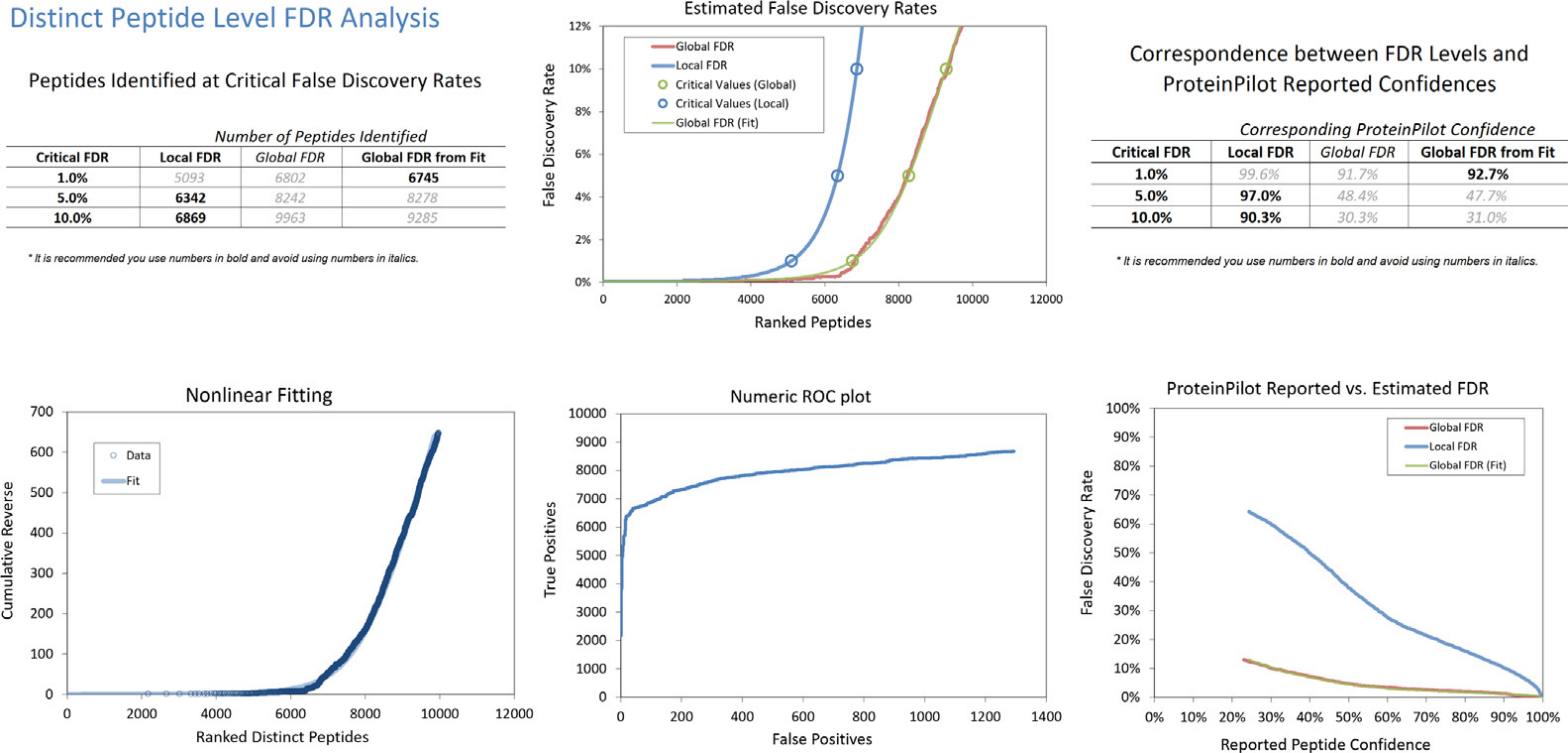
FDR analysis of the combined IDA ion library of tears at peptide level from ProteinPilot software.

Short-sightedness (myopia) is the most common refractive error in the world where approximately 60% of the 12-years-old children in Hong Kong are currently affected [1]. One of the most successful clinical management for myopia is orthokeratology (Ortho-K), which is a therapy that uses custom-made rigid gas permeable contact lenses for overnight wear in young patients [2,3]. Tears from Ortho-k lens wear for 1 month and 3 months (and age-matched controls) were searched using ProteinPilot software (v5.0.1, Sciex Framingham, MA) software against the Uniprot database (Homo Sapiens 26,199) with Paragon Algorithm [4]. A total of 519 non-redundant proteins and 6745 distinct peptides (at 1% FDR) were identified from the combined ion library (Table S1) with FDR analysis at protein level (Fig. 1) and peptide level (Fig. 2). Differentially expressed proteins (1 month and 3 months, compared to their respective age-matched controls) were quantified with SWATH-MS with Oneomics™ platform using fold changes and p-value filtering criteria: Fold change ≥ 1.5-fold change with at least 2 peptides per protein (ion score ≥ 99), P ≤ 0.05, unpaired T-test (Table S2).

## 2 Experimental design, materials, and methods

### 2.1 Tears sample collection

Twelve healthy subjects (aged 18–30 years) were fitted for overnight lens wear while 12 age-matched subjects without lens wear were recruited as control (n = 24 for each time points). Tears were collected using disposable MicroCap® microcapillary tubes after 1 month and 3 months of orthokeratology lens wearing. The protocol of this clinical study was approved by the Human Subjects Ethics Sub-Committee of The Hong Kong Polytechnic University.

### 2.2 Tears protein extraction and digestion for LC-MS/MS

Tears samples collected from the microcapillary tubes were eluted into tubes and the protein concentration was determined using Bradford protein assay. Equal concentration of tears from each patient at each condition (n = 12) was pooled together into 3 biological groups (for 1 month, 3 months and their respective age-matched controls). Thirty micrograms (30 μg) of pooled tear proteins in each group were then homogenized with a lysis buffer (7M Urea, 2M Thiourea, 30mM Tris, 2% CHAPS, 1% ASB14 and protease inhibitor) in a 1:1 volume to buffer ratio under vigorous vortex for 30 seconds, repeated 5 times. The lysed tear samples were then reduced by dithiothreitol at a final concentration of 10 mM for 60 min at 37 °C and alkylated at a final concentration of 40 mM iodoacetamide in the dark for 30 min at room temperature. Acetone precipitation was performed by adding 4× volume of ice-cold acetone overnight at −20 °C, then centrifuge at 21,380×*g* for 30 min at 4 °C. The pellet was then washed with 500 μl of 80% acetone and centrifuged at 21,380×*g* for 10 min at 4 °C. in 50 mM TRIS-HCL (pH 8.5) and was subjected to in-solution digestion with trypsin at 1:25 (enzyme: protein) ratio w/w for 16 hours at 37 °C. Digestion was stopped with 0.5% TFA and was desalted and cleaned-up with C18 SPE HLB column (Waters, USA). Peptide concentration was estimated using Pierce Quantitative Colorimetric Peptide Assay (Thermo) and was calibrated at a final concentration of 0.5 μg/μl. A total of 2 μg of each sample was injected for Mass spectrometry.

### 2.3 Setting of LC-MS/MS

Both IDA and SWATH-acquisitions were acquired using a hybrid Quadrupole Time-of-Flight Triple TOF ® 6600 mass spectrometer (SCIEX). Peptides were loaded on to the trap column (100 μm × 2 cm, C18) by loading buffer (0.1% Formic acid, 2% Acetonitrile in water) at 2 μl min-1 for 15 min and the analytical column (100 μm × 30 cm, Smartube C18, 5 μm) using an Ekisgent 415 nano-LC system. LC separation was under 350 nL min-1 using mobile phase A (0.1% Formic acid, 2% Acetonitrile in water) and B (0.1% formic acid, 98% Acetonitrile in water) with the following gradient: 0–0.5 min: 5%B, 0.5–90 min: 10%B, 90–120 min: 20%B, 120–130 min: 28%B, 130–135 min: 45%B, 135–141 min: 80%B, 141–155 min: 5%B. Peptides were injected into the mass spectrometer with a 10 μm SilicaTip electrospray emitters (New Objective Cat. No. FS360-20-10-N-20-C12). TOF-MS mass scan was set from 350 m/z to 1800 m/z with 250 ms accumulation time, followed by 100 m/z – 1800 m/z for MS/MS scans in high sensitivity mode with 50 ms accumulation time of up to top 50 ion candidates per cycle, ions that exceed the threshold of 125 cps were counted for MS/MS with the charge state between 2 and 4. Rolling collision energy was selected to trigger collision-induced dissociation. For data independent acquisition (DIA, SWATH-MS), the instrument was tuned for a variable isolation window in a looped mode over the mass range of 100 m/z to 1800 m/z scan of 100 overlapping variable windows. An accumulation time of 30 ms was set for each fragment ion resulting a total duty cycle of 3.1s.

### 2.4 Ion library generation for SWATH analysis

Two micrograms tryptic peptide from each of the pool samples were used for the IDA experiment. Combined data from the IDA experiment was used to generate a combined ion library (.group) for SWATH analysis. The data were searched against Homo Sapiens Uniprot database [5] and protein identification (ID) was acquired using ProteinPilot (v5.0.1, Sciex) software with Paragon algorithms with the following settings: Trypsin as the enzyme, cysteine alkylation using iodoacetamide (IAA), thorough search effort and biological modification. The resulting protein pilot file (.group) with 1% false discovery rate (FDR) was used as the ion library for all SWATH file processing and quantification.

### 2.5 SWATH-MS data analysis

Two micrograms of tryptic digested peptides were used for each SWATH injection. Two technical replicates were performed for each of the experimental group (1 month, 3 months and their respective aged matched control). All raw SWATH files (.wiff) were processed with PeakView (v 2.1, Sciex Fragmingham, MA) and uploaded to OneOmics data environment via CloudConnect micro application in PeakView. Normalization of the MS data was calculated based on the Most-Likely-Ratio (MLR) algorithm [6] which calculates sample score and measurement weight for downstream ratio analysis, considering both the biological and technical replicates. For data filtering before quantitation, the following parameters were set: Fold change (≥1.5), p-values (≤0.05) and proteins with at least 2 peptides per protein (ion score ≥ 99), unpaired T-test on the normalized weighted-average peptide areas for each protein across all samples.
